# Horses grazing with cattle have reduced strongyle egg count due to the dilution effect and increased reliance on macrocyclic lactones in mixed farms

**DOI:** 10.1017/S1751731119002738

**Published:** 2020-05

**Authors:** L. Forteau, B. Dumont, G. Sallé, G. Bigot, G. Fleurance

**Affiliations:** 1 Université Clermont Auvergne, INRA, VetAgro Sup, UMR Herbivores, Saint-Genès-Champanelle, France; 2 Institut Français du Cheval et de l’Equitation (IFCE), Pôle développement innovation et recherche, Exmes, France; 3 Institut National de la Recherche Agronomique (INRA), Université François Rabelais Tours, UMR Infectiologie et Santé Publique, Nouzilly, France; 4 Université Clermont Auvergne, AgroParisTech, INRA, Institut national de Recherche en Sciences et Technologies pour l’Environnement et l’Agriculture (Irstea), VetAgro Sup, UMR Territoires, Clermont Ferrand, France

**Keywords:** agroecology, beef cattle, farm survey, health management, nematode

## Abstract

Strongyle infection is an important issue in horse breeding. It impairs horse health and performance, with young horses being the most sensitive. Strongyle control has long relied on the systematic use of chemical treatments. However, expanding anthelmintic resistance among strongyles calls for alternative options. Mixed grazing is assumed to reduce strongyle load on the pasture as the result of a dilution effect. This has been shown in small ruminants grazing with cattle, but the putative benefits of co-grazing between horses and cattle have not yet been evaluated. Here, we conducted field surveys and face-to-face interviews on 44 farms from two contrasted saddle-horse production areas, Normandy and northern Massif Central, to compare equine strongyle management practices between specialized systems and mixed horse-cattle systems. Our goals were (i) to quantify breeders’ awareness of the putative benefits associated with the co-grazing of horses and cattle, (ii) to establish whether mixed farming was associated with different strongyle management strategies and (iii) to test whether strongyle egg excretion was reduced in horses grazed with beef cattle. Every breeder relied on systematic calendar treatments, and only 8 out of the 23 mixed breeders were aware that co-grazing of horses with cattle could be used as part of their strongyle control strategy. Management practices were similar across both systems in Normandy. In Massif Central, mixed breeders formed a distinct cluster from their specialized counterparts: deworming was less frequent and stocking density was higher in mixed farms, while specialized breeders seemed more willing to integrate herd and plot management into control strategies. Faecal egg counts measured in horses from Massif Central were significantly reduced when horses were grazed with cattle. This was the result of an increased reliance on macrocyclic lactones in mixed farms (*P* < 0.01) and a significant dilution effect (*P* < 0.01). When considering a subsample of horses treated with macrocyclic lactones only, young horses grazed with cattle had 50% fewer strongyle eggs excreted in their faeces than horses grazed in equine-only pastures (*P* < 0.01). This is the first evidence of the benefits of mixed grazing with cattle as an alternative to control strongyle infection in horses, although this promising alternative remains largely unknown by horse breeders.

## Implications

Horse breeders are increasingly challenged by drug resistance when controlling strongyle infection. Although largely unknown by mixed breeders, alternate grazing or co-grazing with horses and cattle is assumed to reduce strongyle load in pastures as the result of a dilution effect. Here, we reveal a decrease in strongyle egg excretion in young saddle horses grazing with beef cattle. This dilution effect is likely to decrease treatment frequency and thus veterinary costs and environmental side-effects of drug metabolites on dung beetle assemblages.

## Introduction

Parasitic infection by strongyle nematodes (mostly cyathostomins) is common in grazing horses. High levels of infection affect horse welfare and performance and can eventually lead to death (Giles *et al*., 1985). Cyathostomin infection is indeed associated with enteropathy, leading to protein losses and potentially to horse death when a large number of encysted larvae are released from the mucosa of the large intestine, a phenomenon called ‘larval cyathostomosis’ (Love *et al*., 1999). Young horses between 1 and 4 years are the most sensitive to strongyles, as they are still developing immunity (Love and Duncan, 1992; Lind *et al*., 1999; Kornaś *et al*., 2010).

Strongyle control in saddle horses is classically based on a calendar treatment with anthelmintics, but this practice is currently challenged. First, drug resistance has accumulated in many countries (Relf *et al*., 2014; Sallé *et al*., 2017; Nielsen *et al*., 2018). Second, the metabolites of some of these drugs exert detrimental side-effects on dung beetle assemblages (Sands and Wall, 2018). Therefore, alleviating drug selection pressure on equine strongylid communities should reduce environmental side-effects and decrease associated economic costs (Lester *et al*., 2013; Sallé *et al*., 2015).

An alternative strategy could be to limit the risk of horse infection by co-grazing with another species in order to disrupt parasites’ life cycles by reducing contact between hosts and infective larvae (Waller, 2006; Hoste and Torres-Acosta, 2011). Gastrointestinal parasites indeed exhibit high specificity for their hosts, apart from liver flukes and the nematode species *Trichostrongylus axei*, which infects both horses and cattle. Because of the relative host specificity of parasitic helminths, each grazing species is assumed to act as a dead-end for the parasites infecting members of the other species. The so-called ‘dilution effect’ has been successfully implemented with cattle and sheep, whereby lamb parasite burden was significantly reduced (Marley *et al*., 2006; Brito *et al*., 2013), and with cattle and goats; strongyle egg excretion by Creole kids grazed on irrigated pastures was significantly reduced by sequential grazing with heifers (Mahieu, 2013).

To our knowledge, the putative benefits associated with co-grazing of horses with another species have never been studied. The sole observations available were obtained from an alternate grazing system in the Netherlands. In that case, ponies were either moved to a pasture previously grazed by sheep or stayed on the pasture they had been grazing throughout spring (Eysker *et al*., 1986). The study found a significant reduction of cyathostomin load in ponies grazing after sheep, which was, however, associated with an increased prevalence of *Trichostrongylus axei* (Eysker *et al*., 1986). Because the alternative pasture was mostly grazed by sheep (with occasional but unquantified grazing by ponies) in the previous year, this trial could not soundly evaluate the benefits of species alternation for parasite control in ponies, but rather accounted for the action of moving ponies to a pasture with limited cyathostomin contamination.

In an attempt to evaluate the putative benefits associated with the co-grazing of horses and cattle, we undertook a survey across 46 French farms located in two contrasted French saddle-horse production areas, that is, Normandy and the lowlands of northern Massif Central. Our objectives were (i) to establish if horse breeders integrate herd and grassland management as part of their strongyle control strategy, (ii) to analyse if horse deworming and grazing management differ between mixed horse-cattle and specialized horse farms, and (iii) to quantify strongyle egg excretion in both types of system to determine putative benefits of co-grazing horses with beef cattle.

## Material and methods

### Surveys of management practices at the farm level

The study was conducted on 44 farms producing saddle horses with or without beef cattle that were selected according to three additional criteria: at least three mares were kept on a farm, farms were located in lowlands (i.e., lower than 600 m above sea level) and grasslands represented more than 80% of the agricultural area. Normandy and northern Massif Central were considered as two contrasted case studies: in Normandy, high-level sport horses are bred and grazed on productive grasslands, while in Massif Central, horses are mainly bred for leisure and grazed on less productive grasslands.

Face-to-face interviews were carried out on 23 farms from Normandy (12 mixed and 11 specialized farms) and 21 farms from northern Massif Central (11 mixed and 10 specialized farms). Interviews (1 to 2.5-h long according to farm size and the complexity of pasture management) focused on farmer beliefs and practices related to grazing, plot cleaning and animal health management.

### Grassland management variables

Using collected data, three variables were constructed:

(i) The annual mean stocking rate at the farm level (the sum of the mean annual stocking rate per plot multiplied by plot area, divided by the sum of the grazed and mixed plot areas). To obtain three balanced classes for statistical analyses, annual mean stocking rate was considered low between 0.2 and 0.6 livestock unit (**LU**) per hectare, intermediate between 0.6 and 1.0 LU/ha and high between 1.0 and 1.4 LU/ha (see Table 1 for LU definition). Reducing stocking rate is indeed likely to reduce the risks of strongyle infection by horses (Martins *et al*., 2009).
(ii) The proportion of total grassland area in both cut and grazed management (i.e., mixed as opposed to grazed-only grasslands) was partitioned into three categories: lower than 30%, intermediate (between 30% and 45%) or higher than 45% of grassland area. Grazing horses on mixed grasslands can strongly decrease numbers of infective larvae in pastures (Martin-Rosset, 2015).
(iii) The integration of herd and grassland management practices as part of the strongyle control strategy: *none* when strongyle control only relied on a systematic calendar treatment; *yes_livestock* when additional practices were based on herd management, for example, reducing stocking rate (Waller, 2006); *yes_cleaning* when additional practices were based on pasture management, for example, pasture grinding or mowing before animals grazed pastures to decrease larval density in the sward (Cabaret, 2017) and *yes_livestock_and_cleaning* when both types of practices were combined.

**Table 1:** Livestock unit values used for the different categories of horses and beef cattle. We used values recently actualized to account for liveweight evolution in beef cattle (Inosys Réseaux d’élevage, 2018) and saddle horses (Martin-Rosset, 2015)

Saddle horses		Beef cattle (Charolais breed)	
Mare having foaled	1.20	Cow having calved	1.05
Mare (dry or pregnant < 5 month)	0.71	Cow	0.85
Weaned foal	0.56	Weaned calf	0.40
Yearling	0.89	Young cattle 1 to 2 years old	0.60
Young horse 2 to 3 years old	0.94	Young cattle 2 to 3 years old	0.80
Horse > 3 years old	0.78	Bull	1.00
Stallion	1.00		

### Variables related to deworming strategy

The implemented deworming strategy was also addressed during the interviews. Four variables were covered: (i) the number of anthelmintic treatments administered to young horses (0 to 4 years; considered low between 4 and 9 treatments per horse over 4 years, intermediate between 10 and 13, and high between 14 and 20), or mares (low: 1 to 2 treatments per year; high: 3 to 4); (ii) the strategy used for deworming (systematic, systematic with additional treatments (based on indicators such as diarrhoea, poor coat or body condition), or systematic plus additional treatments based on faecal egg counts (**FECs**)); (iii) the person in charge of parasite control (the breeder, the veterinarian or both) and (iv) the anthelmintic class given to young horses and mares.

The egg reappearance period (**ERP**) indeed varies across anthelmintic classes (Gokbulut *et al*., 2001; Relf *et al*., 2014). To account for egg reappearance in a synthetic way, we considered the proportion of macrocyclic lactones (moxidectin and ivermectin) used for young horses (low: less than 40%; intermediate: 40% to 60%; high: 60% to 90%; almost exclusive: more than 90%) and mares (low: less than 50%; high: 50% to 90%; exclusive: 100%), as macrocyclic lactones have the longest ERPs.

Finally, as the strategy of parasitism control may vary according to the genetic merit of horses, farms were classified into three groups based on French genetic indices of mares currently on the farm in the different equestrian disciplines (jumping, dressage, eventing and endurance). A farm was considered of excellent genetic merit when at least one of the mares was registered with a score higher than +9 for one of the genetic indices. A farm was considered of high genetic merit when at least one of the mares was registered with a positive score for one index. All other farms were considered to produce horses for leisure.

### Strongyle faecal egg counts in horses from mixed and specialized farms

To test whether FEC was reduced in horses from mixed systems, faeces were sampled in autumn in horses at greater risk of infection (18 to 42 months old). For logistical reasons and according to breeders’ willingness to participate, the sampling took place on Massif Central farms only.

Sampled horses were either grazed with cattle (*n* = 23 horses, 6 farms) or alone in a specialized farm (*n* = 23 horses, 5 farms). Fresh individual faeces were collected on the ground and kept at 4°C for less than 48 h; eggs were counted (test sensitivity: 15 eggs/g) at the official local veterinary services (DDCSPP du Puy de Dôme), based on sedimentation and concentration by a flotation technique (Raynaud, 1970).

Sampled horses had been treated with anthelmintic treatments having different ERPs, which induced extra-variance in measured FEC. Recent reports suggest these ERPs have become shorter over the years (Tzelos *et al*., 2017; Molena *et al*., 2018), but current ERPs are unknown in France. In a conservative approach, we chose to correct measured FEC by the last anthelmintic class used according to their original ERPs; that is, 90 and 60 days for moxidectin and ivermectin, respectively; 28 days for pyrantel and 0 days for fenbendazole.

### Statistical analyses

Statistical analyses were performed with R software (The R Core Team, 2019, 3.4.4 version). As variables were all transformed into qualitative variables, a multiple correspondence analysis was conducted followed by hierarchical classification with the FactoMineR package (Lê *et al*., 2008).

To establish whether FEC was significantly associated with the farming system, a linear regression was performed. We built a full model regressing FEC (*n* = 46) on farming system (two classes), the number of treatments (three classes), the proportion of time each horse spent on mixed grasslands, grassland stocking rate and time since the end of the ERP. A variable selection procedure was implemented with the stepAIC() function of the MASS package that retained the farming system, the time since the end of ERP and stocking rate.

In a second analysis, we considered a subsample of 28 horses treated with macrocyclic lactones only. A two-sample *t* test was used to test for equality of FEC means between horses from specialized (*n* = 9) and mixed systems (*n* = 19). For all statistical analyses, effects were considered significant when the *P*-value was lower than 0.05.

## Results

### The putative benefits associated with the co-grazing of horses and cattle were largely overlooked

Face-to-face interviews were also undertaken to evaluate the general beliefs and awareness regarding parasite control management in both mixed and specialized systems. Strongyle control relied on a systematic calendar treatment in all 44 farms surveyed. The most commonly used anthelmintics were fenbendazole (in 36 farms) and macrocyclic lactones (ivermectin, *n* = 33 farms; moxidectin *n* = 22 farms). Additional herd and grassland management practices were implemented and considered as part of breeders’ strongyle control strategy in a total of 18 farms, 11 from Massif Central and 7 from Normandy.

Plot-cleaning practices were the most common (*n* = 14 farms) and included pasture liming (*n* = 5), use of rotary slashers (*n* = 5) or dung-spreading harrows (*n* = 4), avoiding rapidly returning herds to the same pasture (*n* = 3) and removing dung from pastures (*n* = 1). The sum is higher than 14, as some breeders used 2 of these grassland management practices.

Herd management strategies were reported in nine farms, and only eight mixed horse-cattle breeders surveyed were aware that mixed grazing could be used to control strongyle infection in saddle horses. The remaining 15 breeders of that group did not know or believe in any benefit of mixed grazing to decrease horse parasite burden. Reducing stocking rate was reported three times. Two breeders maintained stable group composition, and another used rotational grazing for parasite control.

### Implementation of mixed grazing discriminates between farms in Massif Central but not in Normandy

Following the analysis of breeders’ awareness, we attempted to summarize their strategies in order to establish whether mixed farming systems were associated with particular practices. Multiple correspondence analysis applied to management practice data revealed three clusters separated along two axes explaining 13.0% and 10.6% of total variance, respectively. The first cluster, named Nor, included 22 farms, mainly in Normandy (*n* = 18), two-thirds of which were mixed cattle-horse farms and one-third specialized horse farms. Remaining clusters, named MC-mix and MC-spe, were mostly composed of mixed (70%) or specialized (83%) farms from northern Massif Central, respectively. This suggests that practices were different between mixed and specialized systems in Massif Central, whereas practices were much more similar across systems in Normandy.

Despite variable reliance on macrocyclic lactones in both the MC-mix and MC-spe systems (Table 2), parasite control strategies accounted for significant portions of their discrepancies. Indeed, drug usage was lighter in farms belonging to the mixed cluster (Table 2), while breeders from the specialized cluster were more reliant on veterinarians to decide upon treatment (in 42% of these farms deworming decisions were only made by veterinarians, Table 2). Specialized breeders were also more willing to integrate herd and plot management into control strategy (Table 3), and they mostly decided upon deworming according to certain indicators or FEC (Table 2). These discrepancies may be underpinned by the higher genetic merit of the mares reared in the specialized farms (excellent or high) relative to that from the MC-mix cluster that was more variable (excellent in 40% of the farms but low in the remaining 60%).

**Table 2:** Deworming practices in mixed horse-cattle and specialized horse farms grouped in the three clusters obtained from multiple correspondence analysis and hierarchical clustering on principal components

Deworming practices	Clust-1 Nor (22 farms)	Clust-2 MC-mix (10 farms)	Clust-3 MC-spe (12 farms)	*P*-value
Strategy of horse deworming (in % of farms in which practices were used)				<0.05
Systematic only	0	40	17	
Using indicators	77	60	58	
With FEC	23	0	25	
Number of annual anthelmintic treatments for mares (% of farms)				<0.001
Low (1 to 2)	64	100	17	
High (3 to 4)	36	0	83	
Total number of anthelmintic treatments for 0 to 4 year-old horses (% of farms)				<0.001
Low (4 to 9)	27	90	0	
Intermediate	55	10	50	
High (14 to 20)	18	0	50	
Proportion of macrocyclic lactone treatments for mares (% of farms)				<0.01
Low (<50%)	4	40	25	
High (50% to 90%)	23	30	58	
Exclusive	73	30	17	
Proportion of macrocyclic lactone treatments for young horses (% of farms)				<0.01
Low (<40%)	18	20	42	
Intermediate	14	50	42	
High (60% to 90%)	41	0	16	
Exclusive	27	30	0	
Person in charge of deworming protocol (% of farms)				<0.05
Veterinarian only	4	20	42	
Breeder only	55	70	16	
Both	41	10	42	

FEC = faecal egg counts.

Clust-1 Nor is mostly composed of Normandy farms; Clust-2 MC-mix is mostly composed of mixed farms from northern Massif Central; Clust-3 MC-spe is mostly composed of specialized horse farms from northern Massif Central.

**Table 3:** Grazing management practices in mixed horse-cattle and specialized horse farms grouped in the three clusters obtained from multiple correspondence analysis and hierarchical clustering on principal components

Grazing management factors	Clust-1 Nor (22 farms)	Clust-2 MC-mix (10 farms)	Clust-3 MC-spe (12 farms)	*P*-value
Mean annual stocking rate at farm level (in % of farms)				<0.01
Low (<0.6 LU/ha)	9	20	59	
Intermediate	77	30	33	
High (>1 LU/ha)	14	50	8	
Proportion of total grassland area under cut and grazed management (% of farms)				<0.01
Low (<30%)	50	10	8	
Intermediate	36	80	42	
High (>45%)	14	10	50	
Herd management or plot-cleaning practices used for strongyle control (% of farms)				<0.01
None	77	70	8	
Herd management	9	10	17	
Plot cleaning	5	20	50	
Both are combined	9	0	25	

LU = livestock unit.

Clust-1 Nor is mostly composed of Normandy farms; Clust-2 MC-mix is mostly composed of mixed farms from northern Massif Central; Clust-3 MC-spe is mostly composed of specialized horse farms from northern Massif Central.

The two Massif Central clusters were also contrasted for grassland management. Annual stocking rate was higher in mixed farms. Half of the farms from the MC-mix cluster indeed had annual stocking rates higher than 1 LU/ha, while the proportion of grasslands under both cut and grazed management usually fell in the intermediate class (Table 3). Conversely, nearly 60% of the farms from the MC-spe cluster had an annual stocking rate lower than 0.6 LU per ha, while the proportion of grasslands under both cut and grazed management was the highest of all three clusters (Table 3).

### Horse faecal egg counts is lower in mixed farms

FEC was conducted on 46 horses from mixed and specialized farms to establish whether farming system was impacting horse excretion and what variables underpinned this variation. Our regression model found that time since the end of ERP was significantly driving observed variation in FEC (*F*_*1,42*_ = 11.74, *P* < 0.01) but not stocking rate (*F*_*1,42*_ = 2.61, *P* = 0.11). Farming system was also associated with a significant difference in FEC (*F*_*1,42*_ = 10, *P* < 0.01), with horses from the mixed farming systems excreting half as many eggs than their counterparts from specialized farms.

**Figure 1:**
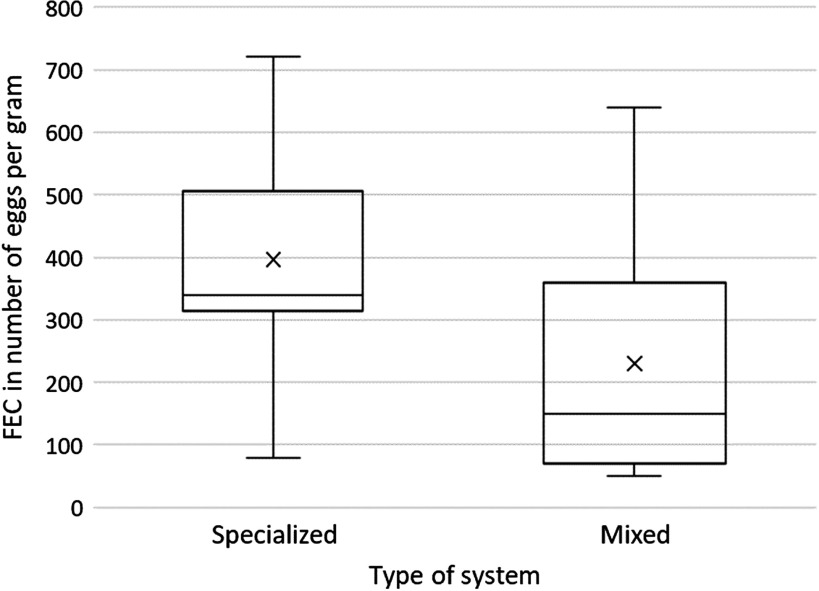
Box and whisker plot of FEC in horses from specialized farms (*n* = 9, mean = 400 epg, median = 340 epg) or grazing with cattle in mixed farms (*n* = 19, mean = 230 epg, median = 150 epg) (*t* test, *P* < 0.01). FEC = faecal egg counts.

A second analysis was performed on a subsample of 28 horses last treated with macrocyclic lactones. It confirmed that young horses grazed with cattle had 50% fewer strongyle eggs excreted in their faeces than horses grazed in equine-only pastures (*P* < 0.01; Figure 1).

## Discussion

The purpose of our study was to compare specialized saddle horse farms with mixed horse-cattle farms and to establish whether any difference in parasite control could be found. The limited awareness of the putative beneficial effects associated with the co-grazing strategy was striking, especially because mixed grazing is a key pillar of integrated parasite management in agroecological grassland-based systems (Dumont *et al*., 2014). These results are consistent with other surveys. In Sweden, mixed grazing was found in 10% of horse farms (Lind *et al*., 2007), while 39% of horse breeders sampled in Rio de Janeiro state (Brazil) indicated they used mixed grazing with ruminants as an alternative to control strongyles and for better pasture management (Martins *et al*., 2009). These results strongly contrast with what has been observed in Irish equine farms, where 71% of respondents were aware of the health benefits of mixed grazing and utilized it effectively to lower strongyle pasture contamination (O’Meara and Mulcahy, 2002). Some of the mixed breeders we surveyed reported grazing horses and cattle on separate pastures because they want to keep mares close to farm buildings but accept grazing suckler cows that require less day-to-day surveillance on more distant pastures. One breeder was also concerned about agonistic interactions between the horses and cattle. Beyond this, a majority of mixed breeders surveyed (15 out of 23) were not aware of the benefits of mixed grazing for parasite management. This stresses the need to pursue the transfer effort of research outputs so that these become available to horse breeders.

This lack of awareness regarding mixed grazing was also associated with the widespread use of fenbendazole, despite high prevalence of resistant strongyle populations in France (Traversa *et al*., 2012; Sallé *et al*., 2017). All breeders also used systematic horse chemical treatment, which confirms previous surveys in Irish (O’Meara and Mulcahy, 2002) and Swedish (Lind *et al*., 2007) horse structures. However, additional preventive strategies like plot-cleaning practices were also implemented in a third of surveyed farms, corroborating findings gathered in Ireland (O’Meara and Mulcahy, 2002) and Sweden (Lind *et al*. 2007). Some of these practices, however, do not provide efficient control of strongyles. This is, for example, seen in the case of pasture liming, reported by five farmers from our survey, which does not provide strongyle control when the annual spreading is applied in March, and in the case of the temporary exclusion of horses from some pastures for decreasing parasite load (reported by three farmers), as it is usually too short to be efficient (Martin-Rosset, 2015).

A key finding from our surveys is that specialized horse breeders from Massif Central seemed more willing than the mixed breeders to integrate herd and plot management into their control strategies. In these farms, the proportion of grasslands under both cut and grazed management was also the highest, and early cuts under mixed management are known to strongly decrease the number of infective larvae in pastures (Martin-Rosset, 2015). Nearly 60% of these farms had an annual stocking rate lower than 0.6 LU/ha, which is likely to reduce the risks of strongyle infection by horses (Martins *et al*., 2009). There was also a high frequency of deworming treatments in these specialized farms, where breeders partly based deworming decisions on indicators. In Sweden, many specialized horse breeders also made additional anthelmintic treatments based on indicators such as the body condition or the presence of worms in faeces (Lind *et al*., 2007). Overall, specialized horse breeders developed more elaborate strategies for controlling strongyle infection in their animals. Their concern may partly result from the higher genetic merit of their mares compared to those in mixed cattle-horse farms in the Massif Central case study. In addition, our observations suggest that deworming practices were much more similar across systems in Normandy, where mare genetic merit was the highest (it was excellent in 77% of the farms and high in the remaining 23%). Normandy breeders strongly relied on macrocyclic lactones and used additional treatments based on indicators such as diarrhoea, poor coat or body condition, or faecal egg counts. A first hypothesis for this discrepancy between the two case studies is that the high genetic merit of their mares may have led Normandy breeders to adopt a risk-adverse strategy that homogenized strongyle management practices.

While our results are thus likely influenced by regional context, they also provide the first indication that the co-grazing of horses and cattle has beneficial effect for equine strongyle control. Our results are consistent with previous observations in ruminant farming, showing that simultaneous grazing by cattle and sheep (Southcott and Barger, 1975; Giudici *et al*., 1999; Marley *et al*., 2006; Brito *et al*., 2013), alternate grazing with cattle and sheep (Rocha *et al*., 2008) or mixed grazing by cattle and goats (Mahieu, 2013) reduce parasitic infection in lambs and goat kids. Because our survey was designed to characterize both types of farming systems with a number of similar characteristics between farms, it was not possible to sample the whole range of equine-to-cattle ratio. Further research is now needed to investigate how the ratio between the two species may influence the contamination of horses by strongyles. Additionally, integrated health management usually combines different practices to control parasitic infection in grassland-based systems (O’Meara and Mulcahy, 2002; Waller, 2006; Dumont *et al*., 2014). Mixed grazing by cattle and horses could, for instance, be combined with the short-term use of tannin-rich plants in the horse diet, as this practice has been shown to impair strongyle egg development into infective larvae (Collas *et al*., 2018). Finally, both specialized and mixed horse breeders were shown to use various herd management and plot-cleaning practices. Further research is needed to provide evidence of their effectiveness in the control of horse strongyles (Martin-Rosset, 2015).

## Conclusion

We provide the first evidence of a decrease in the parasite burdens of young saddle horses grazing the same pastures as cattle in mixed farms, compared with horses grazing alone in specialized systems. This opens a promising alternative for controlling horse parasitic infection that remains largely unknown by horse breeders. Association between horses and cattle at pasture is facilitated through the use of the same type of fencing for the two species, with possible limitations due to injury risks which could be solved through alternate grazing. This suggests a diversity of more sustainable agroecological parasite management strategies in horse farms as alternatives to repeated chemical treatment, which raises serious resistance issues.

## Software and data repository resources

No new software or databases were generated as outcomes of this research.
